# Food environment with high plant-based fat supply is associated with Attention-Deficit/Hyperactivity Disorder (ADHD) protection: a global study with more than 150 countries

**DOI:** 10.3389/fnut.2025.1658228

**Published:** 2025-11-11

**Authors:** Duan Ni, Alistair Senior, David Raubenheimer, Stephen J. Simpson, Ralph Nanan

**Affiliations:** 1 Sydney Medical School Nepean, The University of Sydney, Sydney, NSW, Australia; 2 Nepean Hospital, Nepean Blue Mountains Local Health District, Sydney, NSW, Australia; 3 Charles Perkins Centre, The University of Sydney, Sydney, NSW, Australia; 4 School of Life and Environmental Sciences, The University of Sydney, Sydney, NSW, Australia; 5 Sydney Precision Data Science Centre, The University of Sydney, Sydney, NSW, Australia

**Keywords:** Attention-Deficit/Hyperactivity Disorder (ADHD), fat, plant-based fat, nutrient, diet, food environment

## Abstract

**Background:**

Diet and nutrients are emerging key players in neurological disorders. Attention-Deficit/Hyperactivity Disorder (ADHD) is a major neurodevelopmental disorder on a global scale, affecting children and increasingly being recognized and diagnosed in adult populations. While its aetiology is unclear, it appears to involve a combination of genetic and environmental factors, particularly food environments including diets and nutrients. However, most studies so far have focused on the impacts of individual nutrients or dietary patterns on clinically established ADHD. In contrast, the interactive effects of diets and nutrients and their complex interplay with other factors like socioeconomic status on ADHD prevalence and incidence have so far been overlooked. Here, we aim to systematically interrogate the association between nutrient supply, reflecting the food exposure and dietary environment, socioeconomic status and ADHD disease prevalence and incidence at a global level over time.

**Methods:**

ADHD disease burden data (incidence and prevalence), macronutrient supply and gross domestic product (GDP) were collated from more than 150 countries from 1990 to 2018 and analyzed with nutritional geometric framework generalized additive mixed models (GAMMs).

**Results:**

Modeling results suggested the interactive effects of food environment and socioeconomic status on ADHD. Fat supply, especially plant-based fat supply, is associated with decreased ADHD prevalence and incidence. These associations were conserved across sexes and ages. They were not confounded by the total energy supply.

**Conclusion:**

Globally, fat, particularly plant-based fat supply in food environment correlated with the reduction of ADHD prevalence and incidence, implying its potential protective effects. This is supported by previous reports about the amelioration of ADHD with ketogenic diets. Further in-depth studies are needed to elucidate the underlying mechanistic. This might potentially provide some evidence for future targeted dietary interventions for ADHD prevention.

## Introduction

Diets and nutrients are emerging critical players in neurological pathologies. For example, we have recently shown that a high fat low carbohydrate dietary environment is linked to reduced disease prevalence and incidence of multiple sclerosis and epilepsy globally (1, 2). This prompts us to investigate the effects of diets and nutrients on other neurological disorders.

Attention-Deficit/Hyperactivity Disorder (ADHD) is a common major neurodevelopmental disorder (3, 4), with increasing cases and disease-related mortality globally, and accounting for close to 1% of the disability-adjusted life year (DALY) of global mental disorders (5). ADHD is most prevalent in childhood and exhibits a gender bias toward males (5). Globally, ADHD seems to be substantially impacting Australasia and North America (5). The aetiology and pathophysiology of ADHD are still unresolved but appears to involve both genetic and environmental factors (5, 6). In this context, diets and nutrients have been a focus of interest, but results of these studies are so far inconclusive. It is generally acknowledged that ADHD is related to unhealthy diets. For example, “Western diets” and saturated fat were linked to ADHD aggravation (7, 8), while Mediterranean diets and polyunsaturated fatty acids were reported to improve ADHD symptoms (6, 9–12). However, most studies have primarily focused on the effects of specific foods or diets for ADHD treatment, while overlooking the effects of macronutrients and the possible interactions among nutrients and the broader socioeconomic contexts. Furthermore, previous studies were mostly based on small-scale cohorts, and there is a lack of knowledge about the potential protective effects of dietary exposure and food environments on ADHD at a population level. Considering the emerging global burden of ADHD, insights at a global scale are of great research and clinical interests.

The term “global food and nutritional environment” encompasses the accessibility, quality, quantity, affordability, and source of nutrients and foods available for human consumption. It is emerging as a critical player in determining global health and diseases (13). Global nutrient supply data is one of the upstream determinants of population-wide dietary patterns, and ultimately, individual diets. National nutrient supply has been shown to correlate with average national diet (14, 15), and is thus widely accepted as proxy of the food and nutritional environment, presenting unprecedented opportunities to decipher how food environment might be linked to changes in the global ADHD disease burden. In this context, disentangling the intercorrelatedness of complex data with linear and non-linear effects is challenging, and requires a multi-dimensional approach (16, 17). We previously proposed adopting nutritional geometric framework (NGF) generalized additive mixed models (GAMMs) for such analyses (1, 2, 16–19). NGF is a multi-dimensional analytical tool to systematically elucidate the individual, additive, and interactive effects of various nutritional factors (e.g., 3 macronutrients, protein, carbohydrate and fat) on outcomes of interests, like ADHD prevalence and incidence.

Leveraging NGF GAMMs, we examined the associations between macronutrient supply and ADHD incidence and prevalence at a global scale, also distinguishing the effects of plant- versus animal-based macronutrients. Our analyses will help to direct future diet- and nutrient-targeted research for ADHD management.

## Materials and methods

### Data collection and processing

Data collation and processing were adapted from our previous studies (17, 19) (Supplementary material). ADHD data was obtained from the Global Burden of Disease Study (GBD), in which ADHD is defined as *an externalising disorder, incorporating disability from persistent inattention and/or hyperactivity-impulsivity*. DSMIV-TR (314.0, 314.01) and ICD-10 (F90) diagnostic criteria were used. Macronutrient supply data were collected from the Food and Agriculture Organization Corporate Statistical Database (FAOSTAT, www.fao.org/faostat/en/#home). For protein and fat, their supply data (g/capita/day) were directly obtained from FAOSTAT and converted to supply data of caloric value (kcal/capita/day). For carbohydrate, supply data was calculated by subtracting protein, fat and alcohol supply quantities from the reported total caloric supply (kcal/capita/day), as previously described (13). For plant- versus animal-based nutrients comparison, the corresponding data were obtained from FAOSTAT directly based on FAOSTAT’s classifications. Gross domestic product (GDP) data was from the Maddison project (20).

In brief, analyses were based on global data from 1990 to 2018, which were first filtered for countries or time points with no data record. Where ADHD, macronutrient supply, or GDP data were missing for a given (country, time point) combination, it would be excluded. Resulting data, covering more than 150 countries globally were analyzed with R (Supplementary Figure S1).

### Generalized additive mixed models (GAMMs)

Details of GAMMs were described in (1, 2, 17, 19) Supplementary material. GAMM is a type of multiple regression with similar assumptions to generalized linear models (21–23) and was used to examine the changes in ADHD prevalence and incidence over time and to evaluate the impacts from macronutrient supply. GAMMs account for the nonlinear terms as nonparametric smoothed functions, often in a form of spline. GAMMs provide a flexible manner to estimate the nonlinear associations, which are commonly found in nutritional studies and are gradually receiving more interests, given their implication in both health and diseases (1, 2, 16). Notably, GAMMs also account for a series of confounders, such as GDP in this case, which reflects broader national and socioeconomic development and can strongly influence diet and nutrient availability.

All modeling was run with the *mgcv* package (v1.9–1) (24, 25) in R v4.4.1. They considered the country that the data were based on as a random effect, partly accounting for some inter-country differences in environmental and genetic factors. Models with several variables (e.g., macronutrient supplies, GDP and time) and their different combinations, as well as a null model where only the random effect from the country is considered, are compared. Following our prior works (1, 2, 13, 17, 19), models with multiple variables consider all combinations of the individual, additive and interactive effects among parameters such as macronutrient supply, year and GDP data. The gamma parameter, stipulating the smoothing degrees of the modeled effects was defined as log(n)/2, where n is the number of combinations for countries and years with available data. A Gaussian family with log-link function was used for modeling.

Modeling results were compared using Akaike information criterion (AIC) and the models with the lowest AICs were selected. To examine if our models might be overfitted, latent scale intraclass correlations (ICCs) were computed. ICCs reflect the degree of variance explained by the countries’ random effects. In this context, when comparing two models, if including more parameters leads to a decrease in ICC, this suggests that the parameters help to account for part of the inter-country variance, indicating that their inclusions are meaningful.

GAMMs rely on visual depiction for the interpretation of the smooth terms, because it is not possible to quantify the fitted effects as effect sizes. Selected models were visualized by plotting their predicted values based on the corresponding variables (e.g., macronutrient supplies, etc.) as response surface heatmaps over the nutrient space. Within the modeling surfaces, red reflects higher, while blue indicates lower values. Along the black contour lines, the modeling values are unchanged and the numbers on the lines note the magnitude of the parameters. Visualization was done utilizing *ggplot2* (v3.5.2), *Cairo* (v1.6–2), *gridExtra* (v2.3) packages in R.

## Results

**Figure 1:**
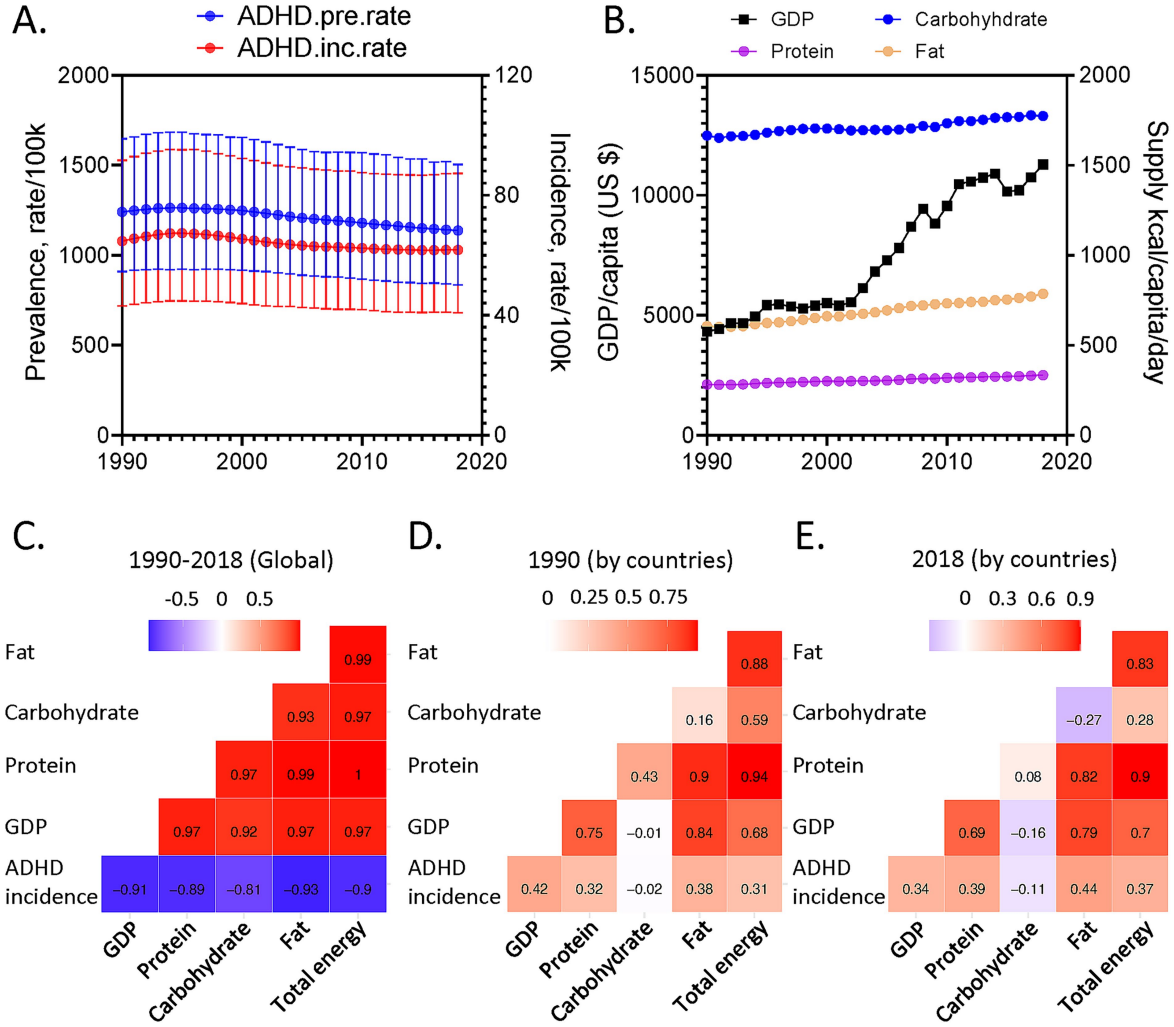
**(A)** Global age-standardized Attention-Deficit/Hyperactivity Disorder (ADHD) prevalence (blue) and incidence rate (red) of both sexes as functions of year. **(B)** Global GDP per capita (in US dollars, black) and supplies of carbohydrate (blue), protein (purple) and fat (brown) as functions of year. **(C–E)** Correlations for global variables from 1990 to 2018 **(C)** and for variables among different countries in 1990 **(D)** and 2018 **(E)**. Correlation coefficients are shown.

Global age-standardized ADHD prevalence and incidence mildly decreased over time (Figure 1A), accompanied by increases in global macronutrient supplies and GDP (Figure 1B). These parameters are interconnected, posing challenges to delineating their detailed associations (Figures 1C–E). Hence, GAMMs, a multi-dimensional modeling tool for global health analysis (13, 17), were deployed to interrogate simultaneously the potential complex interactive effects among various parameters and their links to ADHD. A model considering the interactions between macronutrient supplies and GDP, adding the time effects, was favored (Supplementary material), suggesting the interactive effects of macronutrients and socioeconomic status on ADHD. This model also had a lower latent scale ICC relative to the model only considering the effects of time, GDP, and the countries’ random effects, implying that inclusion of macronutrient parameters helps to explain some of the inter-country differences, justifying their inclusions in our modeling.

**Figure 2:**
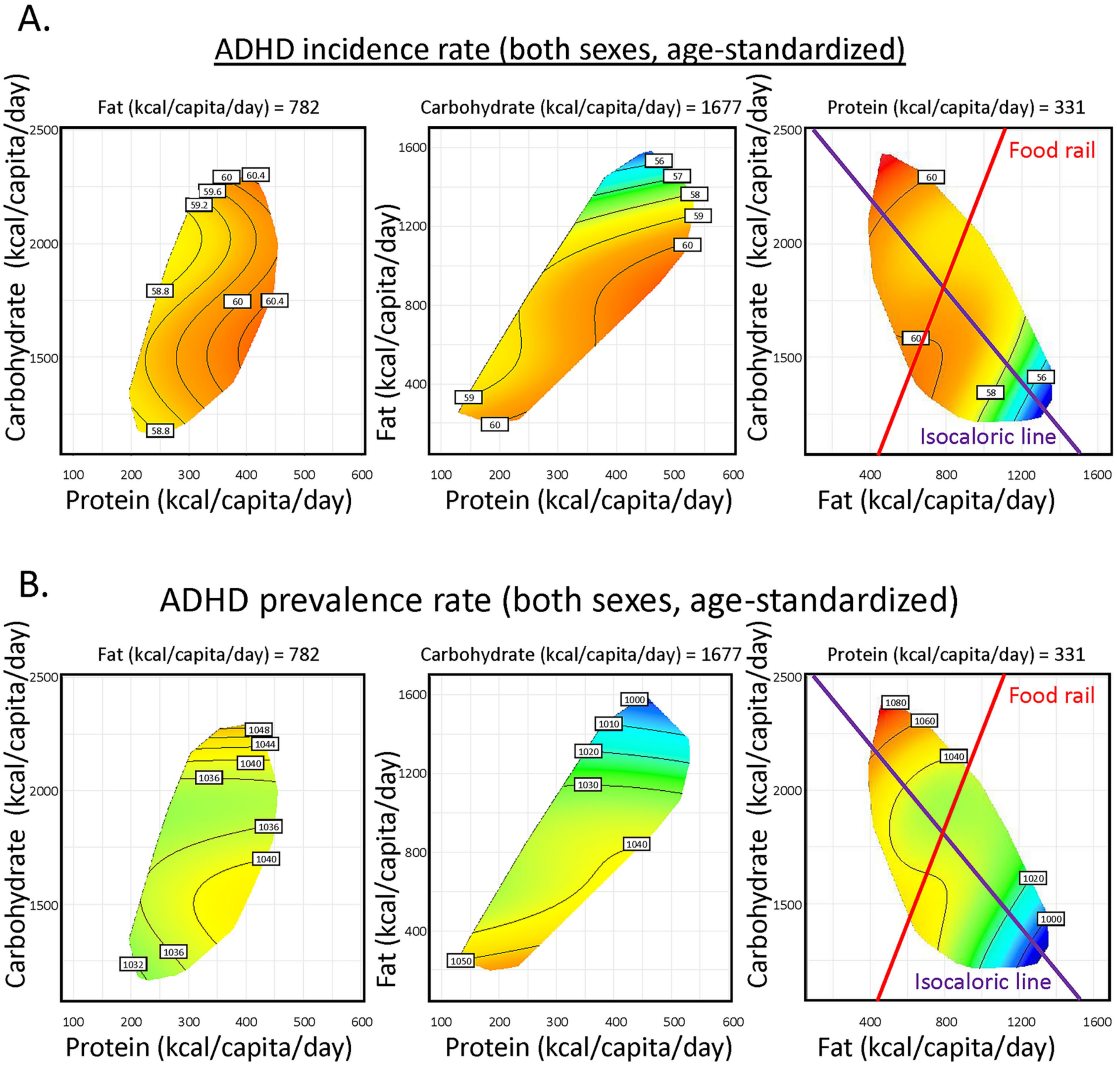
Global association of macronutrient supplies and Attention-Deficit/Hyperactivity Disorder (ADHD) incidence and prevalence. **(A)** Predicted effects of macronutrient supply on age-standardized ADHD incidence rate of both sexes. The left panel shows the effect of carbohydrate and protein supply, the middle shows fat and protein and the right shows carbohydrate and fat supplies. In each panel, the third macronutrient is set at median level (value shown). Surface colors reflect the modeled results, with red indicating higher ADHD incidence and blue indicating lower. **(B)** Predicted effects of macronutrient supply on age-standardized ADHD prevalence rate of both sexes (See Supplementary material for statistics and interpretation).

Results for 2018 was shown as representative (Figure 2A), the most recent year with relatively comprehensive data coverage for more than 150 countries globally. Protein supply minimally influenced ADHD. In contrast, increasing fat supply correlated with lower ADHD incidence (Supplementary Tables S1, S2). The purple vector is an *isocaloric line*, along which the total energy supply is constant, and fat is isocalorically substituted for carbohydrate. Increasing fat:carbohydrate ratio is associated with lowered ADHD incidence. Similar patterns were consistently found for ADHD prevalence (Figure 2B; Supplementary Tables S3, S4) and across genders and ages (Supplementary Figures S2A–C). The red radial is a *food rail*. Along this vector, total energy supply differs, while fat:carbohydrate ratio is constant. Changing total energy supply only mildly impacted ADHD incidence. Importantly, sensitivity tests were performed, where (1) countries with the highest (Australia, Supplementary Figure S3A) or lowest (United Arab Emirates, Supplementary Figure S3B) ADHD incidences were excluded; (2) half of the countries from the dataset were randomly excluded (Supplementary Figure S4). They retrieved similar modeling results to our original findings, suggesting the robustness of our analyses.

**Figure 3:**
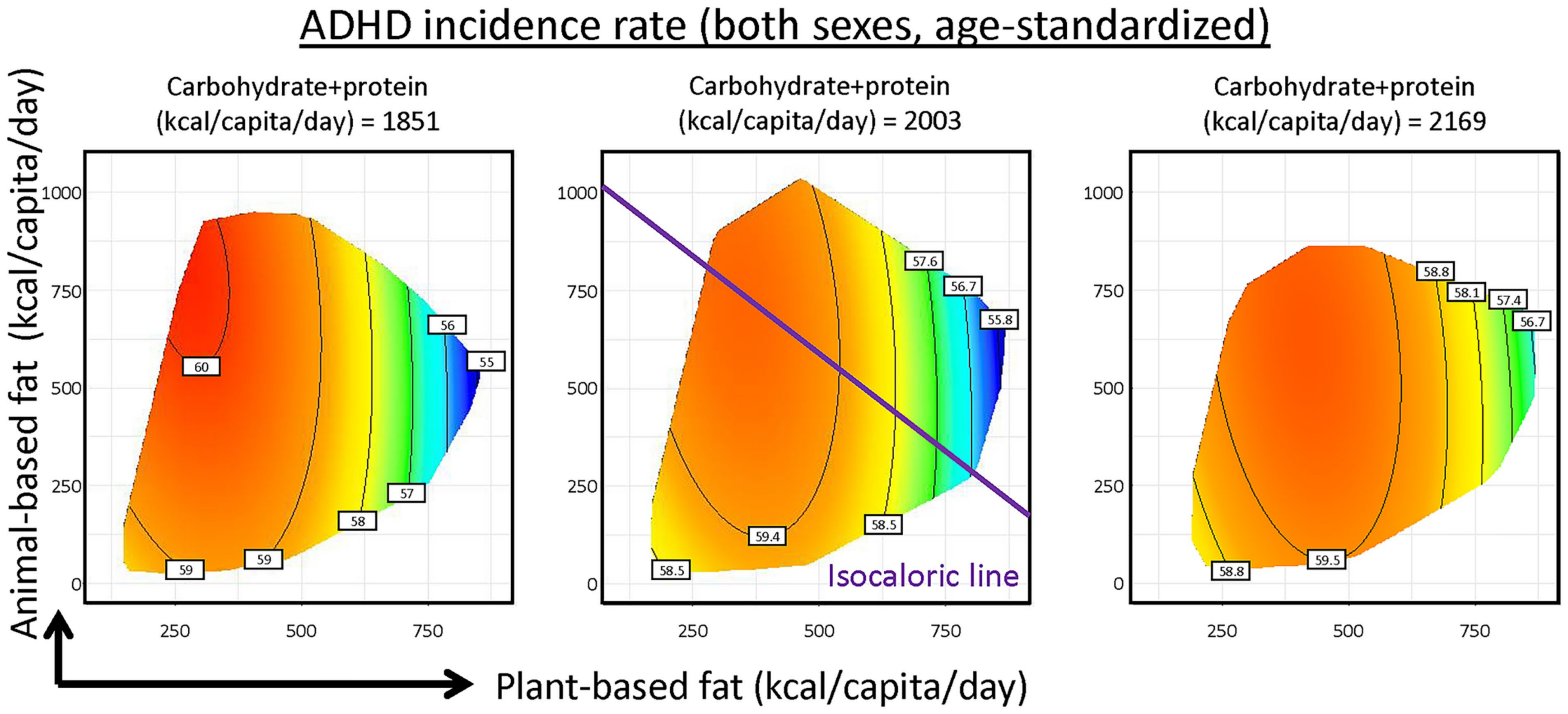
Predicted effects of carbohydrate and protein, animal- and plant-based fat supplies on Attention-Deficit/Hyperactivity Disorder (ADHD) incidence. Mode results were plotted in 2-dimensional nutritional space with plant-based fat supply as *x*-axis and animal-based fat supply as *y*-axis, with supply of carbohydrate and protein held at 25, 50 and 75% quantiles of global data (See Supplementary material for statistics and interpretation).

Effects of fat were further investigated, by sub-setting total fat into plant- (*x*-axis) and animal-based (*y*-axis) fat supplies based on the classifications from FAOSTAT, while combining carbohydrate and protein supplies (Figure 3; Supplementary Tables S5, S6). We showed the effects of plant and animal fats, assuming low, medium and high carbohydrate and protein supplies. Increasing plant-based fat supply associated with decreased ADHD incidence while animal-based fat exhibited limited effects. Along the purple isocaloric line, substituting animal-based fat with plant-based fat while holding total energy content constant is associated with reduced ADHD incidence rate. Surprisingly, for proteins of different sources, plant-based protein supply was linked to increased ADHD incidence (Supplementary Figure S4).

## Discussion

This represents the first study to comprehensively examine global associations between macronutrient supply, as a proxy for food environments, and the incidence and prevalence of ADHD. Our state-of-the-art GAMM analyses provide evidence indicating potential protective effects in ADHD from fat, particularly plant-based fat, with their increasing supplies associated with reduced ADHD incidence and prevalence. This might inform future dietary interventions using plant-based fat for ADHD prevention and/or treatment.

Compared with previous dietary studies on ADHD, our analysis explored its associations with dietary macronutrient supplies and shed light on the potential protective effects of food environment in ADHD. Harnessing unprecedented nutrient supply and global epidemiological data, our study also exceeded any previous similar research regarding sample sizes and the time scales coverage. Our observations applied to both genders and across different age groups, underscoring their robustness. Moreover, we adapted the cutting-edge GAMM approach, thoroughly dissecting the individual, additive and interactive effects of various factors like different macronutrient supplies. This powerful tool might pave the way for future studies related to food environments.

There are still some limitations in our study to be addressed. For example, the diagnostic criteria for ADHD are evolving (26, 27), probably due to the fact that the detailed pathogenesis mechanisms of ADHD are still unresolved but likely to be multifaceted (5, 6). Therefore, in addition to the adjustments included in our present modeling, investigations into other aspects, particularly other environmental factors such as environmental toxins like lead, would be of interests (28). Changes in ADHD diagnostic criteria and a series of other factors, such as fluctuations in ADHD symptoms, differences in health infrastructure, and variations in ADHD reporting compliance, might together contribute to the underestimation of ADHD disease burden in GBD (5). Further validating analyses are needed when methodological issues are addressed in future GBD editions. Similarly, the spatial or temporal autocorrelations within the global data would warrant further explorations. Additionally, more granular global nutrient supply data are not available to allow comparison of simple versus complex carbohydrates, saturated versus non-saturated fats, or associations with supplies of micronutrients like iron, zinc and vitamins, requiring future investigation. Although nutrient supply data provide a proxy for the food environment and dietary exposure, further validation with cohort- or population-based longitudinal studies with more detailed dietary and food intake data, and ADHD data like disease biomarkers, is needed.

Of note, despite the clear patterns we observed, they are correlative and should be interpreted with caution. The causal links between dietary macronutrients and ADHD remains to be further validated and characterized. Previously, systemic investigations on the effects of dietary macronutrients on ADHD prevention and/or treatment are lacking. The most relevant dietary interventions to our results include high fat very low carbohydrate ketogenic diets (29, 30), Mediterranean diets (31, 32), and Mediterranean-DASH Intervention for Neurodegenerative Delay (MIND) diets (33). These have been shown to confer benefits toward ADHD in animal models and small-scale clinical cohorts, but they did not specifically investigate the impacts from macronutrients and their interactions. The fact that plant-based but not animal-based fat was linked to reduced ADHD prevalence and incidence in our analysis, is also in accord with prior studies demonstrating the benefits of vegetable-enriched diets for existing ADHD (12). Similarly, omega-3 polyunsaturated fatty acids, a major component of plant-based fats, have been reported as co-adjuvants for ADHD treatment (34). Nevertheless, further research is required to demonstrate the potential causality, delineate the mechanisms, as well as to interrogate whether plant-based fats potentially exert preventive effects *in utero*, during early infancy and/or directly modify ADHD.

Collectively, our multi-dimensional ecological analyses using NGF GAMM has revealed associations between nutrient supplies and ADHD, highlighting the correlations between the supplies of fat, particularly plant-based fat, and decreased ADHD incidence and prevalence. These findings may potentially provide guide toward future studies to investigate causality and, ultimately, interventions for ADHD management through food and dietary modifications.

## Data Availability

Publicly available datasets were analyzed in this study. This data can be found at: https://www.healthdata.org/research-analysis/gbd, https://www.fao.org/faostat/en/#data/SUA.

## References

[1] Ni D Senior A Raubenheimer D Simpson SJ Nanan R. High-fat and low-carbohydrate dietary environments are linked to reduced idiopathic epilepsy incidence and prevalence. Ann Clin Transl Neurol. (2025) 12:1077–81. doi: 10.1002/acn3.70017, PMID: 40007127 PMC12093339

[2] Ni D Tan J Reyes J Senior AM Andrews C Taitz J . High fat low carbohydrate diet is linked to CNS autoimmunity protection. Adv Sci (Weinh). (2025) 12:e2412236. doi: 10.1002/advs.202412236, PMID: 40150860 PMC12165102

[3] Salari N Ghasemi H Abdoli N Rahmani A Shiri MH Hashemian AH . The global prevalence of ADHD in children and adolescents: a systematic review and meta-analysis. Ital J Pediatr. (2023) 49:48. doi: 10.1186/s13052-023-01456-1, PMID: 37081447 PMC10120242

[4] Polanczyk G de Lima MS Horta BL Biederman J Rohde LA. The worldwide prevalence of ADHD: a systematic review and metaregression analysis. Am J Psychiatry. (2007) 164:942–8. doi: 10.1176/ajp.2007.164.6.942, PMID: 17541055

[5] Cortese S Song M Farhat LC Yon DK Lee SW Kim MS . Incidence, prevalence, and global burden of ADHD from 1990 to 2019 across 204 countries: data, with critical re-analysis, from the global burden of disease study. Mol Psychiatry. (2023) 28:4823–30. doi: 10.1038/s41380-023-02228-3, PMID: 37684322

[6] Faraone SV Bellgrove MA Brikell I Cortese S Hartman CA Hollis C . Attention-deficit/hyperactivity disorder. Nat Rev Dis Primers. (2024) 10:11. doi: 10.1038/s41572-024-00495-0, PMID: 38388701

[7] Colter AL Cutler C Meckling KA. Fatty acid status and behavioural symptoms of attention deficit hyperactivity disorder in adolescents: a case-control study. Nutr J. (2008) 7:8. doi: 10.1186/1475-2891-7-8, PMID: 18275609 PMC2275745

[8] Morandini HAE Watson P Stewart RM Wong JWY Rao P Zepf FD. Implication of saturated fats in the aetiology of childhood attention deficit/hyperactivity disorder - a narrative review. Clin Nutr ESPEN. (2022) 52:78–85. doi: 10.1016/j.clnesp.2022.10.004, PMID: 36513489

[9] Del-Ponte B Quinte GC Cruz S Grellert M Santos IS. Dietary patterns and attention deficit/hyperactivity disorder (ADHD): a systematic review and meta-analysis. J Affect Disord. (2019) 252:160–73. doi: 10.1016/j.jad.2019.04.061, PMID: 30986731

[10] Martin SM I Sanz Rojo S Gonzalez Cosano L Conty De La Campa R Garicano Vilar E Blumenfeld Olivares JA. Impulsiveness in children with attention-deficit/hyperactivity disorder after an 8-week intervention with the Mediterranean diet and/or omega-3 fatty acids: a randomised clinical trial. Neurologia (Engl Ed). (2022) 37:513–23. doi: 10.1016/j.nrleng.2019.09.009 34656505

[11] Sinn N Bryan J. Effect of supplementation with polyunsaturated fatty acids and micronutrients on learning and behavior problems associated with child ADHD. J Dev Behav Pediatr. (2007) 28:82–91. doi: 10.1097/01.DBP.0000267558.88457.a5, PMID: 17435458

[12] Pinto S Correia-de-Sa T Sampaio-Maia B Vasconcelos C Moreira P Ferreira-Gomes J. Eating patterns and dietary interventions in ADHD: a narrative review. Nutrients. (2022) 14:14. doi: 10.3390/nu14204332, PMID: 36297016 PMC9608000

[13] Andrews CJ Raubenheimer D Simpson SJ Senior AM. Associations between national plant-based vs animal-based protein supplies and age-specific mortality in human populations. Nat Commun. (2025) 16:3431. doi: 10.1038/s41467-025-58475-1, PMID: 40210635 PMC11986065

[14] Remans R Wood SA Saha N Anderman TL DeFries RS. Measuring nutritional diversity of national food supplies. Glob Food Secur. (2014) 3:174–82. doi: 10.1016/j.gfs.2014.07.001

[15] Del Gobbo LC Khatibzadeh S Imamura F Micha R Shi P Smith M . Assessing global dietary habits: a comparison of national estimates from the FAO and the global dietary database. Am J Clin Nutr. (2015) 101:1038–46. doi: 10.3945/ajcn.114.087403, PMID: 25788002 PMC4409685

[16] Senior AM Legault V Lavoie FB Presse N Gaudreau P Turcot V . Multidimensional associations between nutrient intake and healthy ageing in humans. BMC Biol. (2022) 20:196. doi: 10.1186/s12915-022-01395-z, PMID: 36050730 PMC9438070

[17] Senior AM Nakagawa S Raubenheimer D Simpson SJ. Global associations between macronutrient supply and age-specific mortality. Proc Natl Acad Sci USA. (2020) 117:30824–35. doi: 10.1073/pnas.2015058117, PMID: 33199593 PMC7720154

[18] Senior AM Raubenheimer D Couteur DGL Simpson SJ. The geometric framework for nutrition and its application to rodent models. Annu Rev Anim Biosci. (2024) 13:389–410. doi: 10.1146/annurev-animal-111523-102327 39546416

[19] Ni D Senior AM Raubenheimer D Simpson SJ Macia L Nanan R. Global associations of macronutrient supply and asthma disease burden. Allergy. (2024) 79:1989–91. doi: 10.1111/all.16067, PMID: 38372164

[20] Inklaar R de Jong H Bolt J Van Zanden JL “Rebasing ‘Maddison’: new income comparisons and the shape of long-run economic development” in GGDC Research Memorandum (Groningen Growth and Development Center), (2018). Vol. GD174.

[21] Diederich A. Generalized additive models.: an introduction with. J Math Psychol. (2007) 51:339–9.

[22] Verbeke T. Generalized additive models: an introduction with R. J Roy Stat Soc A. (2007) 170:262. doi: 10.1111/j.1467-985X.2006.00455_15.x

[23] Wood SN Goude Y Shaw S. Generalized additive models for large data sets. J Royal Statis. Soc. Series C. (2015) 64:139–55. doi: 10.1111/rssc.12068

[24] Wood SN. Fast stable restricted maximum likelihood and marginal likelihood estimation of semiparametric generalized linear models. J R Stat Soc Series B Stat Methodol. (2011) 73:3–36. doi: 10.1111/j.1467-9868.2010.00749.x

[25] Wood SN. Generalized additive models: An introduction with R Chapman and Hall/CRC, New York (2017). Available online at: https://www.taylorfrancis.com/books/mono/10.1201/9781315370279/generalized-additive-models-simon-wood

[26] Rigler T Manor I Kalansky A Shorer Z Noyman I Sadaka Y. New DSM-5 criteria for ADHD - does it matter? Compr Psychiatry. (2016) 68:56–9. doi: 10.1016/j.comppsych.2016.03.008, PMID: 27234183

[27] Sibley MH Kuriyan AB. DSM-5 changes enhance parent identification of symptoms in adolescents with ADHD. Psychiatry Res. (2016) 242:180–5. doi: 10.1016/j.psychres.2016.05.036, PMID: 27288736

[28] Moore S Paalanen L Melymuk L Katsonouri A Kolossa-Gehring M Tolonen H. The association between ADHD and environmental chemicals-a scoping review. Int J Environ Res Public Health. (2022) 19:19. doi: 10.3390/ijerph19052849, PMID: 35270544 PMC8910189

[29] Liu Y Yang C Meng Y Dang Y Yang L. Ketogenic diet ameliorates attention deficit hyperactivity disorder in rats via regulating gut microbiota. PLoS One. (2023) 18:e0289133. doi: 10.1371/journal.pone.0289133, PMID: 37585373 PMC10431618

[30] Garner S Davies E Barkus E Kraeuter AK. Ketogenic diet has a positive association with mental and emotional well-being in the general population. Nutrition. (2024) 124:112420. doi: 10.1016/j.nut.2024.112420, PMID: 38669832

[31] Darabi Z Vasmehjani AA Darand M Sangouni AA Hosseinzadeh M. Adherence to Mediterranean diet and attention-deficit/hyperactivity disorder in children: a case control study. Clin Nutr ESPEN. (2022) 47:346–50. doi: 10.1016/j.clnesp.2021.11.014, PMID: 35063225

[32] Rios-Hernandez A Alda JA Farran-Codina A Ferreira-Garcia E Izquierdo-Pulido M. The Mediterranean diet and ADHD in children and adolescents. Pediatrics. (2017) 139:139. doi: 10.1542/peds.2016-2027, PMID: 28138007

[33] Bayranj Z Fotros D Sohouli MH Rohani P Eslahi M Ferdosi S . The relation between MIND diet with odds of attention-deficit/hyperactivity disorder in Iranian children: a case-control study. Child Neuropsychol. (2025) 31:331–45. doi: 10.1080/09297049.2024.2375493, PMID: 38975687

[34] Chang JP Su KP Mondelli V Pariante CM. Omega-3 polyunsaturated fatty acids in youths with attention deficit hyperactivity disorder: a systematic review and meta-analysis of clinical trials and biological studies. Neuropsychopharmacology. (2018) 43:534–45. doi: 10.1038/npp.2017.160, PMID: 28741625 PMC5669464

