# Supplementary material for: Food environment with high plant-based fat supply is associated with Attention-Deficit/Hyperactivity Disorder (ADHD) protection: a global study with more than 150 countries
Source: Front Nutr. 2025 Nov 11;12:1658228. doi: 10.3389/fnut.2025.1658228 (PMC12644920; doi:10.3389/fnut.2025.1658228)
Supplement: Supplementary file 1 [file Data_Sheet_1.PDF]

Supplementary Information

Interpretation of modelling surfaces

Details for modelling surface interpretation are adapted from previous publications [1-3]. In brief, using RStudio (v4.2.2.), Attention-Deficit/Hyperactivity Disorder (ADHD) disease burden data were analyzed with generalized additive mixed models (GAMMs) prediction [4, 5]. For analysis of the supplies of three macronutrients, results were mapped as response surfaces based on nutrient axes for three macronutrients, protein, carbohydrate and fat. For analysis of fat types, modelling results were projected on responses with plant-based fat on the x-axis and animal-based fat on the y-axis, while holding carbohydrate and protein supply at a range of constants (i.e., 25%, 50% (median) and 75% quantiles of the global supply of different countries).

Within the modelling surfaces, red reflects higher values, while blue implies lower ones. Along the black contour lines, the modelling values are unchanged and the numbers on the lines note the magnitude of the parameters. The purple line is an isocaloric line, along which the total energy supply from macronutrients is unchanged but fat is substituted for carbohydrate isocalorically. The red line is a food rail. Carbohydrate:fat ratio is held constant along it, but the total macronutrient energy supply is altered.

Statistics for the modelling analysis are provided in Supplementary Tables. When the modelling analysis is significant, the effect of macronutrient supply on the modelling values (i.e., ADHD disease burden) can be deduced from the modelling surfaces.

Supplementary Methods

Data collection and processing

Data collation and processing were adapted from previous studies [1, 3]. ADHD data was obtained from the Global Burden of Disease Study (GBD). ADHD is defined as *an externalising disorder, incorporating disability from persistent inattention and/or hyperactivity-impulsivity*. DSMIV-TR (314.0, 314.01) and ICD-10 (F90) diagnostic criteria were used. As described [1], macronutrient supply data and gross domestic product (GDP) data were collected from the Food and Agriculture Organization Corporate Statistical Database (FAOSTAT, www.fao.org/faostat/en/#home) and the Maddison project [6] respectively.

Analyses were based on data from 1990 to 2018 with relatively comprehensive data coverage. Countries or time points with no data record were filtered and the resulting data spanning from 1990 to 2018 covering more than 150 countries, covering all continents, were further analyzed with R (Figure S1).

Generalized additive mixed models (GAMMs)

Details of GAMMs were described in [1, 3]. In brief, GAMMs were used to model the changes in ADHD burden over time and evaluate the impacts from macronutrient supply and GDP. GAMMs are based on assumptions similar to generalized linear models. They take into account the nonlinear terms as nonparametric smoothed functions, often in a form of spline, and provide a flexible manner to estimate the nonlinear associations. All modelling was carried out with the *mgcv* package and its "gam" function [4, 5]. All models considered the country that the data were based on as a random effect. The gamma parameter, implying the smoothing degrees of the modelled effects was defined as log(n)/2, where n is the number of combinations for countries and years with available data. A Gaussian family with log-link function was used for modelling.

Several different predictor variables and their different combinations as well as a null model where only the random effect from the country is considered are compared. Models with multiple variables consider all combinations of the individual, additive and interactions among parameters like macronutrient supply, year and GDP data. Macronutrient supply was modelled as a three-dimensional spline, and year and GDP data were modelled as one-dimensional cubic-regression splines utilizing the "s()" smooth function in *mgcv* package. The interactions between smooth terms on different scales such as macronutrient supply and year were modelled with the "te()" tensor product smooth function from *mgcv* package.

Modelling results were compared using Akaike information criterions (AICs) and the model with the lowest AIC was selected [7].

Codes for analysis are available on GitHub: https://github.com/Nidane/Nutrient-ADHD.

Supplementary Tables

Supplementary Tables S1-4 are GAMMs estimates. For parametric terms the model estimates and associated standard errors (SE) and test statistics are presented. For non-parametric smooth terms, the estimated and reference degrees of freedom (reflected by edf (effective degrees of freedom), sumEDF, Ref.df (reference degrees of freedom)) are shown as well as their test statistics. Smooth terms were fitter with either the standard smooth function “s()” or a tensor product smooth “te()” in the *mgcv* package. Tensor product smoothing was utilized where terms exist on different units (for example, nutrient supply and year). Country was included as a random effect using the smooth function “s()”. The model family and implemented link function is stated.

Supplementary Table S1. Relative fit of generalized additive mixed models (GAMMs) testing the predictors for age-standardized ADHD incidence rate in both sexes. Gamma is the degrees of freedom inflation factor. Dev means the deviance explained. AIC = Akaike information criterion. GDP = gross domestic product. Delta is the differences between AICs of models and the minimum AIC. sumEDF reflects the degrees of freedom of the models. Macronutrient supply was modelled as a three-dimensional thin-plate spline. (related to Figure 2A)

GAM	Gamma	Dev	AIC	Delta	Weights	sumEDF	Latent scale ICC	Formula
1	3.855439	99.76844	17867.64	1633.088	0	157.8517	0.07110039	1 + s(Country, bs="re")
2	3.855439	99.77226	17813.08	1578.518	0	167.6359	0.1091079	s(protein.kcal, carb.kcal, fat.kcal, k=k_nut) + s(Country, bs="re")
3	3.855439	99.78785	17481.19	1246.629	1.98567010450277e-271	160.0696	0.07651745	s(Year, k=10, bs="cr") + s(Country, bs="re")
4	3.855439	99.79119	17420.92	1186.361	2.42622252865814e-258	165.3298	0.1474496	s(GDP, k=10, bs="cr") + s(Country, bs="re")
5	3.855439	99.79188	17415.36	1180.801	3.91213781832251e-257	169.955	0.1395347	s(protein.kcal, carb.kcal, fat.kcal, k=k_nut) + s(Year, k=10, bs="cr") + s(Country, bs="re")
6	3.855439	99.7939	17374.45	1139.895	2.9853797841001e-248	171.3127	0.09342107	s(protein.kcal, carb.kcal, fat.kcal, k=k_nut) + s(GDP, k=10, bs="cr") + s(Country, bs="re")
7	3.855439	99.79369	17371.53	1136.97	1.28866084850942e-247	167.5382	0.3238746	s(Year, k=10, bs="cr") + s(GDP, k=10, bs="cr") + s(Country, bs="re")
8	3.855439	99.82545	16670.65	436.089	2.01589199378044e-95	190.2406	0.1486297	te(protein.kcal, carb.kcal, fat.kcal, Year, bs=c("tp", "cr"), d=c(3,1), k=c(k_nut, 7)) + s(Country, bs="re")
9	3.855439	99.84142	16262.38	27.8253	9.07427843328068e-07	200.3362	0.120957031	te(protein.kcal, carb.kcal, fat.kcal, GDP, bs=c("tp", "cr"), d=c(3,1), k=c(k_nut, 7)) + s(Country, bs="re")
10	3.855439	99.80421	17161.24	926.679	5.94592930369434e-202	179.2737	0.137424918	te(Year, GDP, k=10) + s(Country, bs="re")
11	3.855439	99.83117	16539.13	304.5756	7.28191457923261e-67	198.9599	0.2385499	te(protein.kcal, carb.kcal, fat.kcal, Year, bs=c("tp", "cr"), d=c(3,1), k=c(k_nut, 7)) + s(GDP, k=10, bs="cr") + s(Country, bs="re")
12	3.855439	99.84247	16234.56	0	0.999999	201.3021	0.1528618	te(protein.kcal, carb.kcal, fat.kcal, GDP, bs=c("tp", "cr"), d=c(3,1), k=c(k_nut, 7)) + s(Year, k=10, bs="cr") + s(Country, bs="re")
13	3.855439	99.8091	17068.62	834.0669	7.66797594645443e-182	189.414	0.1478469	te(Year, GDP, k=10) + s(protein.kcal, carb.kcal, fat.kcal, k=k_nut) + s(Country, bs="re")

Supplementary Table S2. Estimated effects of macronutrient supply by time and GDP per capita on age-standardized ADHD incidence rate. Gaussian-GAMM, log-link function. (related to Figure 2A)

**Parametric coefficients**				
	**Estimate**	**Std. Error**	**t value**	**Pr(>|t|)**
**(Intercept)**	4.0736	0.0304	134	<2e-16
**Approximate significance of smooth terms**				
	**edf**	**Ref.df**	**F**	**p-value**
**te(protein.kcal,carb.kcal,fat.kcal,GDP)**	42.36	47.14	29.62	<2e-16
**s(Year)**	1.00	1.00	25.87	6.2e-07
**s(Country)**	157.94	158.00	11586.14	<2e-16
R-sq.(adj) = 0.998 Deviance explained = 99.8%				
GCV = 2.9772 Scale est. = 2.1242 n = 4465				

Supplementary Table S3. Relative fit of generalized additive mixed models (GAMMs) testing the predictors for age-standardized ADHD prevalence rate in both sexes. Gamma is the degrees of freedom inflation factor. Dev means the deviance explained. AIC = Akaike information criterion. GDP = gross domestic product. Delta is the differences between AICs of models and the minimum AIC. sumEDF reflects the degrees of freedom of the models. Macronutrient supply was modelled as a three-dimensional thin-plate spline. (related to Figure 2B)

GAM	Gamma	Dev	AIC	Delta	Weights	sumEDF	Latent scale ICC	Formula
1	3.855439	99.89688	40445.25	1582.066	0	157.929	0.0005091742	1 + s(Country, bs="re")
2	3.855439	99.89899	40372.59	1509.402	0	167.7235	7.245557e-04	s(protein.kcal, carb.kcal, fat.kcal, k=k_nut) + s(Country, bs="re")
3	3.855439	99.90688	39994.02	1130.839	2.76338100413053e-246	160.0603	5.509092e-04	s(Year, k=10, bs="cr") + s(Country, bs="re")
4	3.855439	99.90842	39929.74	1066.557	2.51202423930582e-232	164.9309	7.742974e-04	s(GDP, k=10, bs="cr") + s(Country, bs="re")
5	3.855439	99.90916	39903.58	1040.393	1.2063282842829e-226	169.9863	9.414105e-04	s(protein.kcal, carb.kcal, fat.kcal, k=k_nut) + s(Year, k=10, bs="cr") + s(Country, bs="re")
6	3.855439	99.90991	39875.29	1012.106	1.67489804144539e-220	174.425	8.803691e-04	s(protein.kcal, carb.kcal, fat.kcal, k=k_nut) + s(GDP, k=10, bs="cr") + s(Country, bs="re")
7	3.855439	99.91038	39838.16	974.9703	1.94035545393585e-212	167.4321	2.751238e-03	s(Year, k=10, bs="cr") + s(GDP, k=10, bs="cr") + s(Country, bs="re")
8	3.855439	99.92188	39267.4	404.2108	1.68557157288367e-88	188.7435	1.000393e-03	te(protein.kcal, carb.kcal, fat.kcal, Year, bs=c("tp", "cr"), d=c(3,1), k=c(k_nut, 7)) + s(Country, bs="re")
9	3.855439	99.92671	39006.57	143.3835	7.32258381342056e-32	200.7571	1.812985e-03	te(protein.kcal, carb.kcal, fat.kcal, GDP, bs=c("tp", "cr"), d=c(3,1), k=c(k_nut, 7)) + s(Country, bs="re")
10	3.855439	99.9172	39522.55	659.361	6.6309877932303e-144	186.4748	1.223476e-03	te(Year, GDP, k=10) + s(Country, bs="re")
**11**	**3.855439**	**99.92367**	**39177.01**	**313.8291**	**7.12633457799124e-69**	**195.3426**	**1.772099e-03**	**te(protein.kcal, carb.kcal, fat.kcal, Year, bs=c("tp", "cr"), d=c(3,1), k=c(k_nut, 7)) + s(GDP, k=10, bs="cr") + s(Country, bs="re")**
**12**	**3.855439**	**99.9291**	**38863.19**	**0**	**1**	**203.2088**	**1.850335e-02**	**te(protein.kcal, carb.kcal, fat.kcal, GDP, bs=c("tp", "cr"), d=c(3,1), k=c(k_nut, 7)) + s(Year, k=10, bs="cr") + s(Country, bs="re")**
13	3.855439	99.91922	39433.8	570.6129	1.238742172783e-124	197.1993	2.223329e-03	te(Year, GDP, k=10) + s(protein.kcal, carb.kcal, fat.kcal, k=k_nut) + s(Country, bs="re")

Supplementary Table S4. Estimated effects of macronutrient supply by time and GDP per capita on age-standardized ADHD prevalence rate. Gaussian-GAMM, log-link function. (related to Figure 2B)

**Parametric coefficients**				
	**Estimate**	**Std. Error**	**t value**	**Pr(>|t|)**
**(Intercept)**	6.9389	0.5043	13.76	<2e-16
**Approximate significance of smooth terms**				
	**edf**	**Ref.df**	**F**	**p-value**
**te(protein.kcal,carb.kcal,fat.kcal,GDP)**	44.087	48.940	26.15	<2e-16
**s(Year)**	1.122	1.232	43.05	<2e-16
**s(Country)**	158.000	158.000	27227.68	<2e-16
R-sq.(adj) = 0.999 Deviance explained = 99.9%				
GCV = 474.44 Scale est. = 337.3 n = 4465				

Supplementary Table S5. Relative fit of generalized additive mixed models (GAMMs) testing the predictors for age-standardized ADHD incidence rate in both sexes. Gamma is the degrees of freedom inflation factor. Dev means the deviance explained. pbf = plant-based fat supply. carb_prot = carbohydrate and protein supply. abf = animal-based fat supply. AIC = Akaike information criterion. GDP = gross domestic product. Delta is the differences between AICs of models and the minimum AIC. sumEDF reflects the degrees of freedom of the models. Macronutrient supply (plant- and animal-based fats and carbohydrate and protein) was modelled as a three dimensional thin-plate spline. (related to Figure 3)

GAM	Gamma	Dev	AIC	Delta	Weights	sumEDF	Latent scale ICC	Formula
1	3.855439	99.76844	17867.64	2022.136	0	157.8517	0.07110039	1 + s(Country, bs="re")
2	3.855439	99.77581	17742.72	1897.214	0	167.6289	1.352135e-01	s(pbf.kcal, carb_prot.kcal, abf.kcal, k=k_nut) + s(Country, bs="re")
3	3.855439	99.78785	17481.19	1635.677	0	160.0696	7.651745e-02	s(Year, k=10, bs="cr") + s(Country, bs="re")
4	3.855439	99.79119	17420.92	1575.409	0	165.3298	1.474496e-01	s(GDP, k=10, bs="cr") + s(Country, bs="re")
5	3.855439	99.79299	17390.87	1545.365	0	169.6712	1.555469e-01	s(pbf.kcal, carb_prot.kcal, abf.kcal, k=k_nut) + s(Year, k=10, bs="cr") + s(Country, bs="re")
6	3.855439	99.79506	17349.34	1503.827	0	171.2812	1.129832e-01	s(pbf.kcal, carb_prot.kcal, abf.kcal, k=k_nut) + s(GDP, k=10, bs="cr") + s(Country, bs="re")
7	3.855439	99.79369	17371.53	1526.018	0	167.5382	3.238746e-01	s(Year, k=10, bs="cr") + s(GDP, k=10, bs="cr") + s(Country, bs="re")
8	3.855439	99.84861	16039.26	193.7514	8.46085812691349e-43	192.4324	1.758665e-01	te(pbf.kcal, carb_prot.kcal, abf.kcal, Year, bs=c("tp", "cr"), d=c(3,1), k=c(k_nut, 7)) + s(Country, bs="re")
9	3.855439	99.85481	15874.89	29.38286	4.1647851491474e-07	203.618	2.013374e-01	te(pbf.kcal, carb_prot.kcal, abf.kcal, GDP, bs=c("tp", "cr"), d=c(3,1), k=c(k_nut, 7)) + s(Country, bs="re")
10	3.855439	99.80421	17161.24	1315.727	1.96547598662957e-286	179.2737	0.137424918	te(Year, GDP, k=10) + s(Country, bs="re")
11	3.855439	99.85176	15957.29	111.7778	5.3425550952716e-25	198.3622	1.710523e-01	te(pbf.kcal, carb_prot.kcal, abf.kcal, Year, bs=c("tp", "cr"), d=c(3,1), k=c(k_nut, 7)) + s(GDP, k=10, bs="cr") + s(Country, bs="re")
12	3.855439	99.85586	15845.51	0	1	205.0184	2.062225e-01	te(pbf.kcal, carb_prot.kcal, abf.kcal, GDP, bs=c("tp", "cr"), d=c(3,1), k=c(k_nut, 7)) + s(Year, k=10, bs="cr") + s(Country, bs="re")
13	3.855439	99.80942	17060.2	1214.695	1.7074825617386e-264	188.9548	1.550540e-01	te(Year, GDP, k=10) + s(pbf.kcal, carb_prot.kcal, abf.kcal, k=k_nut) + s(Country, bs="re")

Supplementary Table S6. Estimated effects of macronutrient supply (plant- and animal-based fats and carbohydrate and protein) by time and GDP per capita on age-standardized ADHD incidence rate. Gaussian-GAMM, log-link function. (related to Figure 3)

**Parametric coefficients**				
	**Estimate**	**Std. Error**	**t value**	**Pr(>|t|)**
**(Intercept)**	4.07357	0.04008	101.6	<2e-16
**Approximate significance of smooth terms**				
	**edf**	**Ref.df**	**F**	**p-value**
**te(pbf.kcal,carb_prot.kcal,abf.kcal,GDP)**	45.466	49.951	37.79	<2e-16
**s(Year)**	1.583	1.964	16.52	8.41e-07
**s(Country)**	157.969	158.000	12081.77	<2e-16
R-sq.(adj) = 0.998 Deviance explained = 99.9%				
GCV = 2.7456 Scale est. = 1.9454 n = 4465				

Supplementary Figures

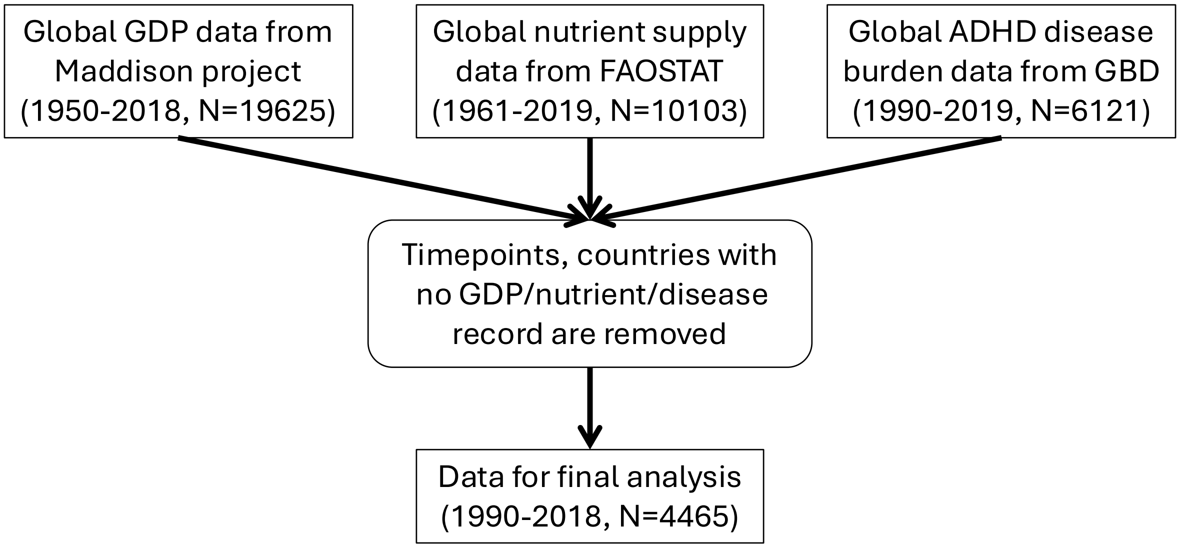

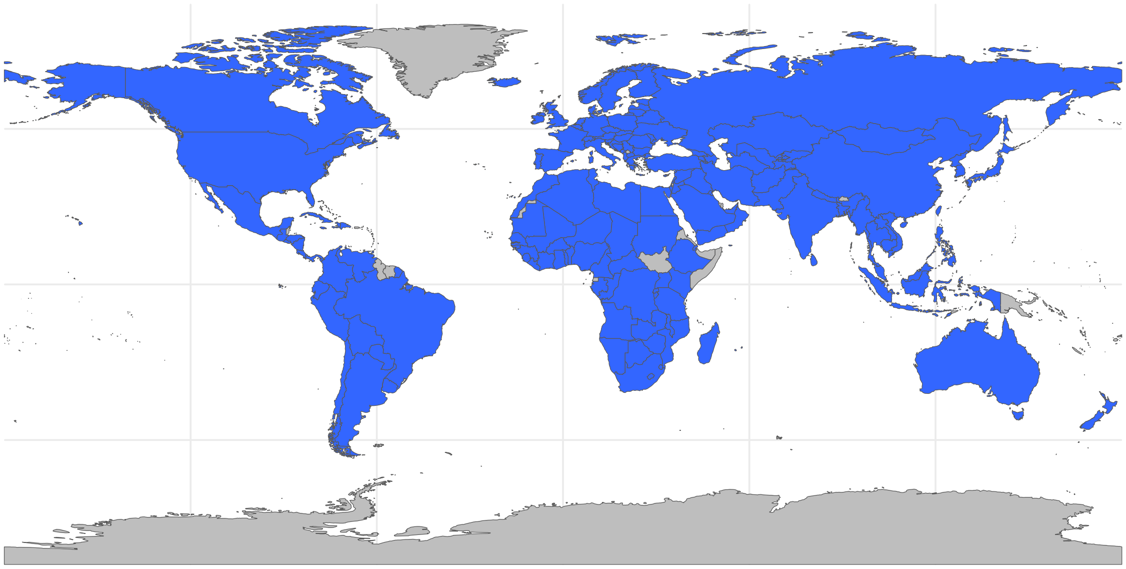

Supplementary Figure S1. An overview of the 159 countries included in the analysis for 2018 around the globe. Countries included in the analysis are highlighted in blue.

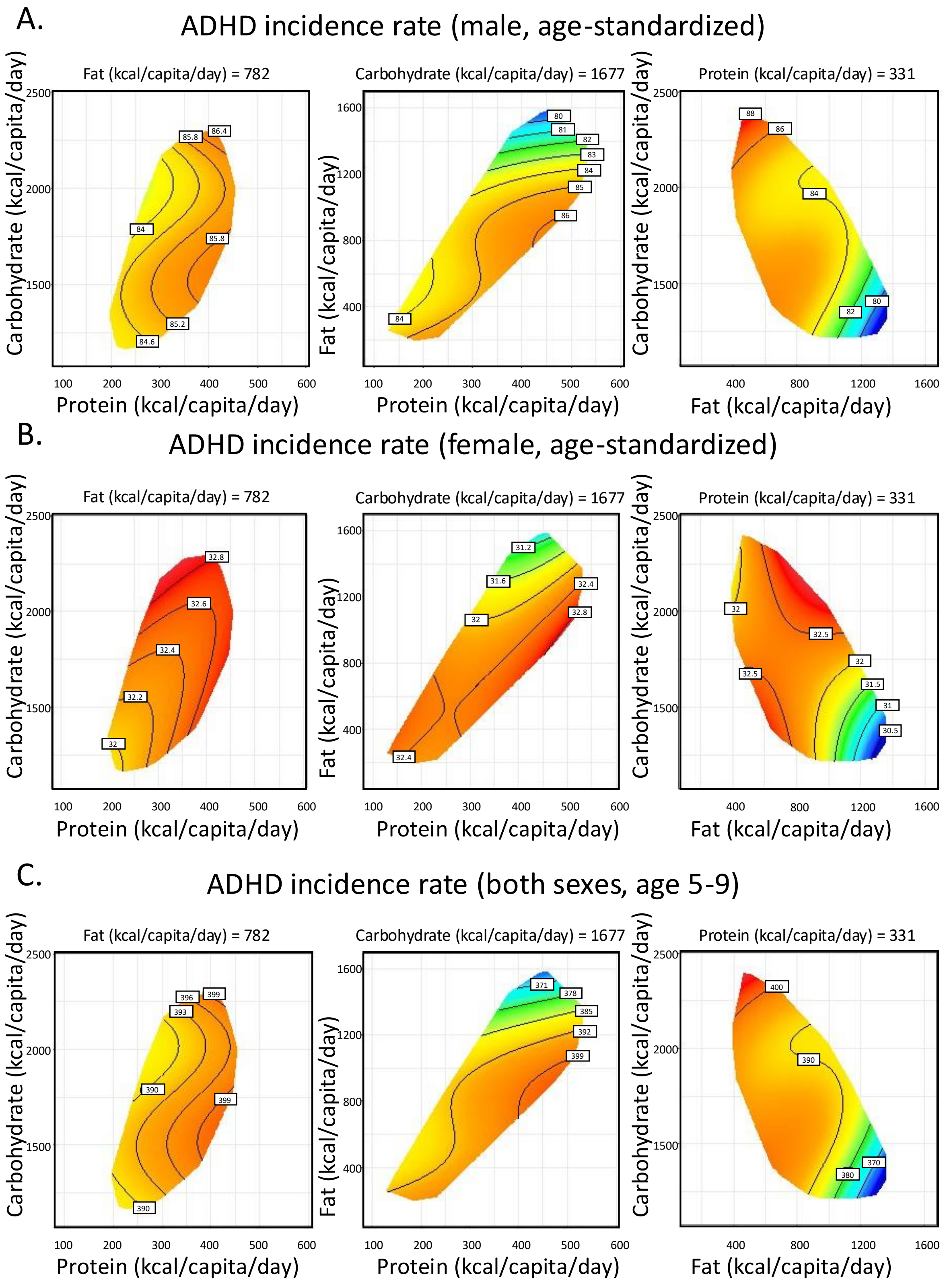

Supplementary Figure S2. Predicted effects of macronutrient supply on age-standardized ADHD incidence rate of male (**A**) and female (**B**), and 5-9 years old ADHD incidence rate of both sexes (**C**).

A. ADHD incidence rate (both sexes, age-standardized)
Excluding Australia

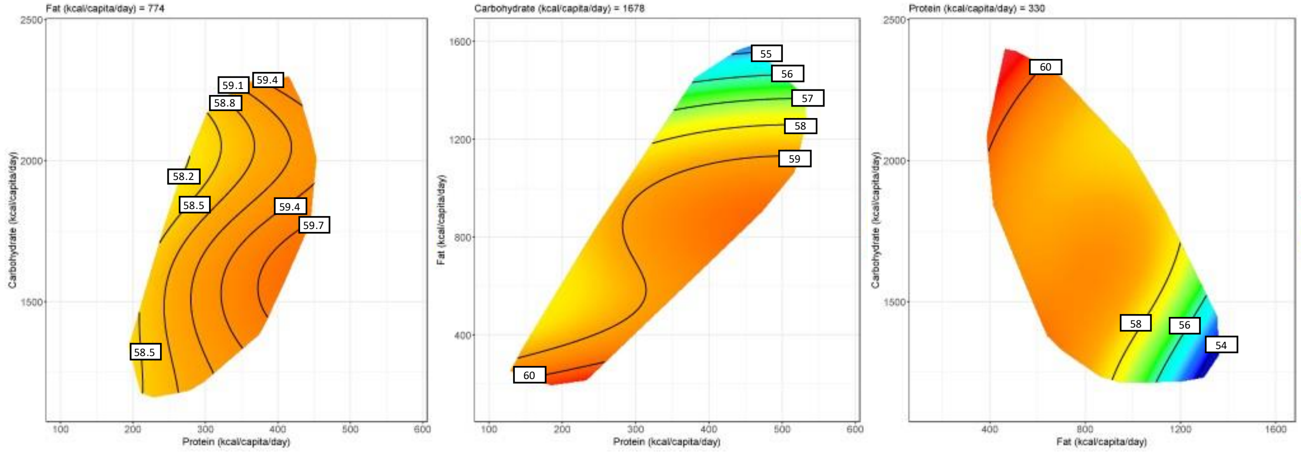

B. ADHD incidence rate (both sexes, age-standardized)
Excluding United Arab Emirates

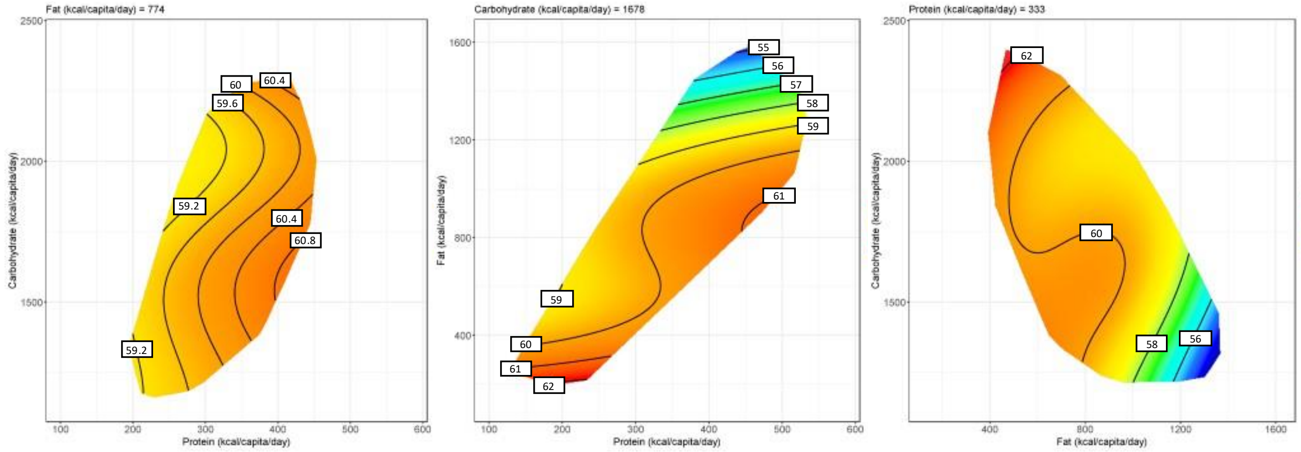

Supplementary Figure S3. Predicted effects of macronutrient supply on age-standardized ADHD incidence rate of both sexes when excluding countries with the highest (Australia, **A**) or lowest (United Arab Emirates, **B**) ADHD incidence rates.

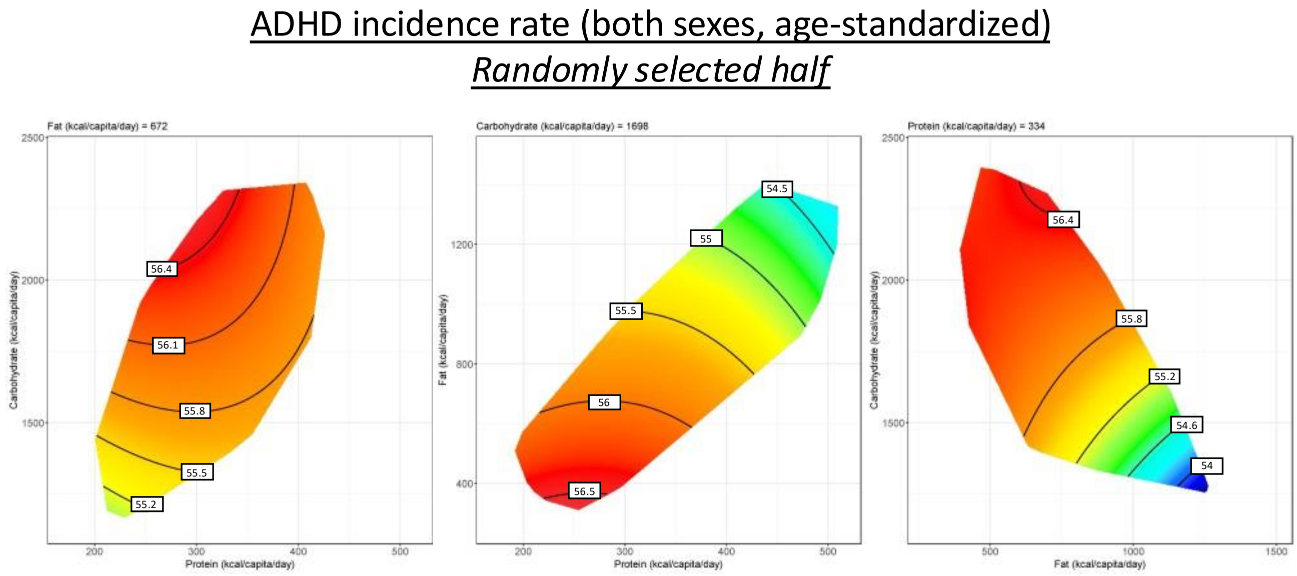

Supplementary Figure S4. Predicted effects of macronutrient supply on age-standardized ADHD incidence rate of both sexes when randomly excluding half of the countries within the dataset.

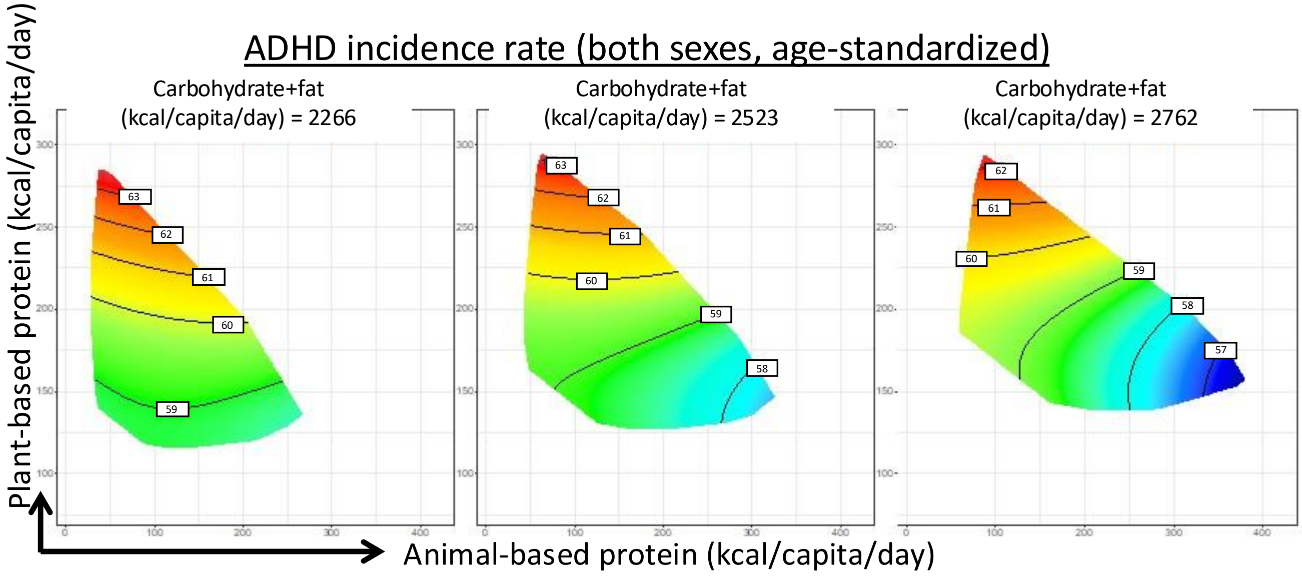

Supplementary Figure S5. Predicted effects of carbohydrate and fat, animal- and plant-based protein supplies on Attention-Deficit/Hyperactivity Disorder (ADHD) incidence.

References

1. Senior AM, Nakagawa S, Raubenheimer D, Simpson SJ: **Global associations between macronutrient supply and age-specific mortality**. *Proc Natl Acad Sci U S A* 2020, **117**(48):30824-30835.

2. Wali JA, Ni D, Facey HJW, Dodgson T, Pulpitel TJ, Senior AM, Raubenheimer D, Macia L, Simpson SJ: **Determining the metabolic effects of dietary fat, sugars and fat-sugar interaction using nutritional geometry in a dietary challenge study with male mice**. *Nat Commun* 2023, **14**(1):4409.
3. Ni D, Senior AM, Raubenheimer D, Simpson SJ, Macia L, Nanan R: **Global associations of macronutrient supply and asthma disease burden**. *Allergy* 2024.
4. Wood SN: **Generalized Additive Models: An Introduction with R**: Chapman and Hall/CRC; 2017.
5. Wood SN: **Fast stable restricted maximum likelihood and marginal likelihood estimation of semiparametric generalized linear models**. *J Roy Stat Soc B* 2011, **73**:3-36.
6. Jutta Bolt RI, Herman de Jong, and Jan Luiten van Zanden: **Rebasing 'Maddison': New income comparisons and the shape of long-run economic development**. In., vol. GD-174. GGDC Research Memorandum: Groningen Growth and Development Center; 2018.
7. Akaike H: **Information theory and an extension of the maximum likelihood principle** In: *Second International Symposium on Information Theory.* Budapest; 1973.
